# Changes in the Arabidopsis RNA-binding proteome reveal novel stress response mechanisms

**DOI:** 10.1186/s12870-019-1750-x

**Published:** 2019-04-11

**Authors:** Claudius Marondedze, Ludivine Thomas, Chris Gehring, Kathryn S. Lilley

**Affiliations:** 1 Cambridge Centre for Proteomics, Cambridge Systems Biology Centre, and Department of Biochemistry, University of Cambridge, Tennis Court Road, Cambridge, CB2 1GA UK; 2 Division of Biological and Environmental Sciences and Engineering, King Abdullah University of Science and Technology, 23955-6900 Thuwal, Kingdom of Saudi Arabia; 3 Department of Chemistry, Biology and Biotechnology, University of Perugia, 74 Borgo XX Giugno, 06121 Perugia, Italy; 4 Present Address: Laboratoire de Physiologie Cellulaire et Végétale, Université Grenoble Alpes, CEA/DRF/BIG, INRA UMR1417, CNRS UMR5168, 38054 Grenoble Cedex 9, France; 5 Present Address: HM.Clause, rue Louis Saillant, 26802 Portes-lès-Valence, France

**Keywords:** *Arabidopsis thaliana*, Drought stress, Proteomics, Mass spectrometry, mRNA interactomics, RNA-binding proteins, Systems analysis

## Abstract

**Background:**

RNA-binding proteins (RBPs) are increasingly recognized as regulatory component of post-transcriptional gene expression. RBPs interact with mRNAs via RNA-binding domains and these interactions affect RNA availability for translation, RNA stability and turn-over thus affecting both RNA and protein expression essential for developmental and stimulus specific responses. Here we investigate the effect of severe drought stress on the RNA-binding proteome to gain insights into the mechanisms that govern drought stress responses at the systems level.

**Results:**

Label-free mass spectrometry enabled the identification 567 proteins of which 150 significantly responded to the drought-induced treatment. A gene ontology analysis revealed enrichment in the “RNA binding” and “RNA processing” categories as well as biological processes such as “response to abscisic acid” and “response to water deprivation”. Importantly, a large number of the stress responsive proteins have not previously been identified as RBPs and include proteins in carbohydrate metabolism and in the glycolytic and citric acid pathways in particular. This suggests that RBPs have hitherto unknown roles in processes that govern metabolic changes during stress responses. Furthermore, a comparative analysis of RBP domain architectures shows both, plant specific and common domain architectures between plants and animals. The latter could be an indication that RBPs are part of an ancient stress response.

**Conclusion:**

This study establishes mRNA interactome capture technique as an approach to study stress signal responses implicated in environmental changes. Our findings denote RBP changes in the proteome as critical components in plant adaptation to changing environments and in particular drought stress protein-dependent changes in RNA metabolism.

**Electronic supplementary material:**

The online version of this article (10.1186/s12870-019-1750-x) contains supplementary material, which is available to authorized users.

## Background

RNA-binding proteins (RBPs) determine RNA fate from synthesis to decay and are increasingly recognized as critical post-transcriptional gene regulators. RBPs bind mRNAs through RNA-binding domains (RBDs) and consequently affect RNA availability for processing and translation [1] essential for stimulus-specific responses [2]. Remarkably, in the animal systems it was noted that modifying the expression pattern or mutating RBPs and/or their target binding sites influence alternative splicing events and can trigger diseases such as neurological disorders and cancers [3–5]. Additionally, transcriptional arrest inducing stress granule formation in response to stresses such as low oxygen, oxidative and heat stresses has been observed [6–10]. Stress granules are cytoplasmic foci formed from cytoplasmic aggregates of non-translated messenger ribonucleoproteins and are described as sites of mRNA storage, sorting and triage. However, they are yet to be fully characterized in plants and other systems.

Systems level detection of the RNA-binding proteome (RBPome) has been made possible by the use of the interactome capture technology and has yielded genome-wide mRNA interactomes in several species [11–17] and they have revealed high degree of similarity between mammalian cells and yeasts [13], as well as plants [12, 18, 19] suggesting an ancient origin. Interactome capture involves in vivo fixing of proteins to their target mRNAs by UV crosslinking followed by purification of mRNA-protein complexes through affinity capture of polyadenylated RNA and then analyzing interacting proteins by tandem mass spectrometry (MS/MS). This technique has a great advantage over other crosslinking techniques based on chemical fixation in that it generates covalent linkages between physically interacting proteins and mRNAs in vivo [20, 21]. It also permits time resolved isolation of RBPs allowing characterization of targeted developmental and physiological states of cellular systems. Furthermore, it has been established that cellular reactions to stress signals compel tight regulation of gene expression including the timely up-regulation of genes encoding for specific stress-responsive factors. However, the respective stress responsive RBPome remain yet to be established. Therefore, we set out to determine whether a defined abiotic stress induces an RBP response signature and did so using a response to drought stress in Arabidopsis as an experimental test system. We firstly established the interactome capture technique as an approach to study stress response, in particular drought responsive RBPome. We argue that changes in the latter will afford insights into the mechanisms that govern metabolic changes during stress and our results would afford a unique systems view RBP changes. Finally, we suggest that stress-induced RBPs may be an evolutionarily conserved mechanism governing post-transcriptional responses to stress.

## Results and discussion

Here we used *Arabidopsis thaliana* cell suspension cultures (ecotype Columbia-0) to obtain and characterize the plant stress RBPome and gain insight into mechanisms that govern stress responses at the systems level. Three biological replicates were treated with 40% (*v*/v) PEG, a dehydration-inducing agent to mimic drought stress, collected samples at 1 h and 4 h and measured ABA levels.

### Abscisic acid assay

**Fig. 1:**
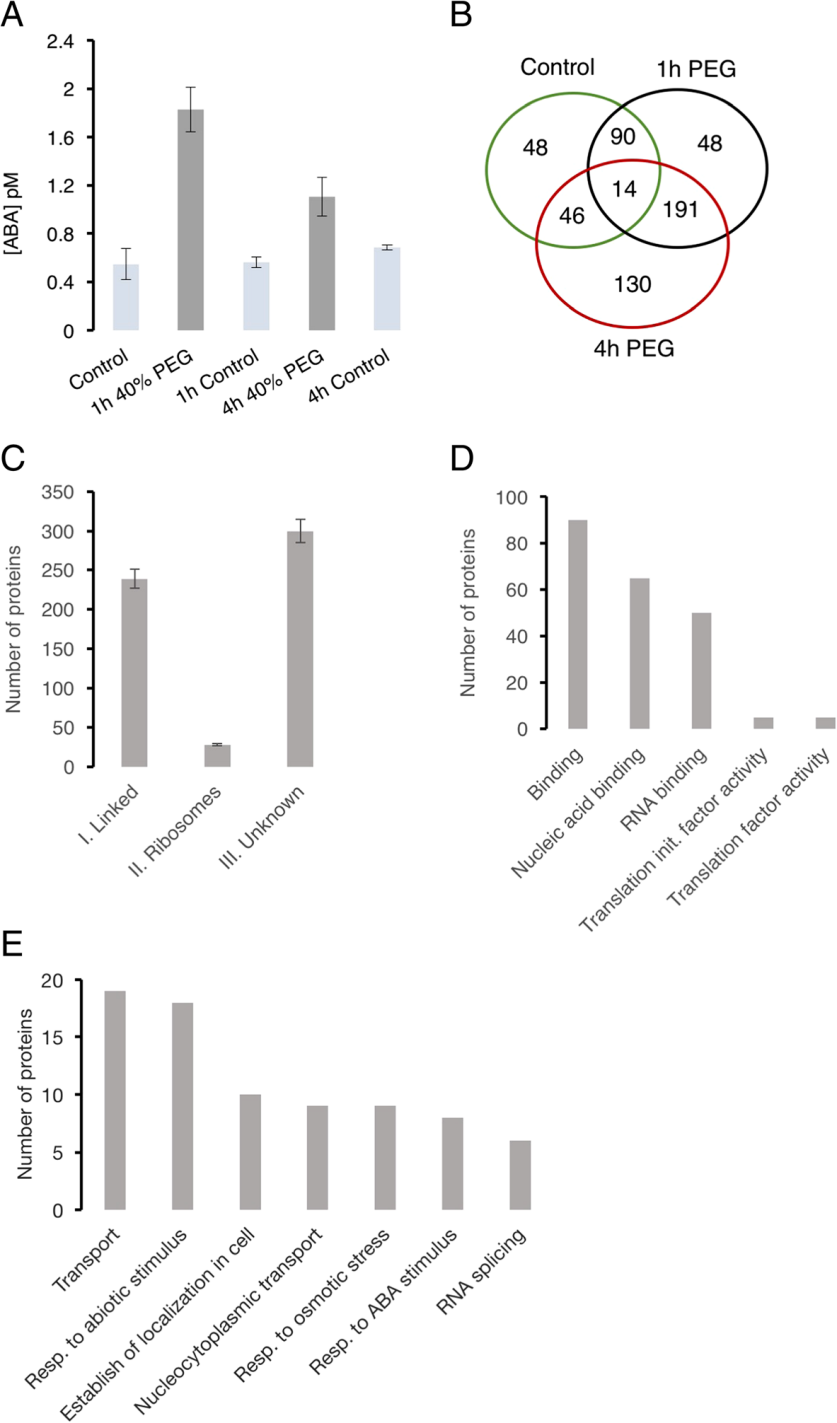
Abscisic acid assay, drought stress responsive RNA-binding proteome (RBPome) and systems classification of drought stress response RNA-binding proteins in *Arabidopsis thaliana*. **a** Abscisic acid assay performed using the Phytodetek® ABA Immunoassay kit showing significant (*p*-value ≤0.05) increases in ABA after drought stress treatment on Arabidopsis cell suspension cultures. **b** Distribution of the RBPome before and after treatments at 1 h and 4 h. **c** Classification of drought stress responsive proteins based on three categories: category 1 proteins linked to RNA biology, category II ribosomal proteins and category III proteins with unknown RNA biology or RNA-binding domains. The bars are standard deviation error bars signifying whether a protein is present in at least two biological replicates. **d**-**e** Gene ontology (GO) molecular function (**d**) and biological processes (**e**) enrichment analyses of the significantly enriched (150 proteins) RBPs linked to drought stress responses

To confirm whether the treatment of cell suspension cultures with 40% (*v*/v) PEG was sufficient to induce or mimic drought stress, we performed an ABA assay using the Phytodetek® ABA Immunoassay kit. A rapid increase in ABA levels at 1 h and 4 h after treatment as compared to the control samples was observed (Fig. 1a). We noted a three-fold increase in ABA at 1 h and a 1.5-fold at 4 h, which is consistent with the induction of drought stress (Fig. 1a). This further validated that exposure to drought stress signals a rapid cellular signal that leads to an increase in the hormonal levels of ABA, a canonical stress marker for drought or water dehydration stress.

### Identification of RBPs responsive to drought stress

Mass spectrometry identified 1408 proteins of which 567 proteins showed specific time-dependent responses to drought stress and these represent the drRBPome. Within the 567 responsive proteins, 178 proteins were detected either at 1 h or 4 h after treatment, 191 proteins were detected at both 1 h and 4 h after treatment while 48 proteins were consistently detected only in the control samples or rather could not be detected upon exposure to stress (Fig. 1b, Additional file 1: Table S1). It is important to note that these time-dependent transient changes that occurred as result of drought stress were considered only when the proteins were detected consistently in all the three biological replicates used in this study. This group of RBPs represent proteins that are present or absent only in the stress-treated samples. The remaining 150 significantly (*p*-value ≤0.05) altered proteins (Fig. 1b, Additional file 1: Table S1) represent the drought stress responsive RBPs (drRPBs). The drRBPs contain the ribosomal protein S15A (AT1G07770), ILITYHIA (AT1G64790) and ABA hypersensitive 1 (CBP80, AT2G13540) that significantly increase in abundance (log_2_-fold change ≥1.5, *p*-value ≤0.05) (Additional file 1: Table S1) at both 1 h and 4 h after treatment. In contrast, the abundance of RNA-binding protein (AT3G15010), flowering time control protein (AT4G16280), hyaluronan (AtRGGA, AT4G16830), co-chaperone GrpE family protein (AT4G26780), glycine-rich RNA-binding protein 8 or cold, circadian rhythm and RNA-binding protein 1 (GR-RBP8, AT4G39260) and nuclear transport factor 2 (NTF2, AT5G43960) decrease. Most of these proteins have previously been linked to drought and/or ABA responses, for example CBP80 and AtRGGA whose role as RBPs have been proposed to be important for a proper response to osmotic stress [22–25]. AtRGGA gene expression was observed to increase in seedlings following a prolonged exposure to either ABA or PEG [22]. This is consistent with the view that these RBPs operate as post-transcriptional modulators of the drought stress response signaling.

Next we assessed the changes at the proteome level of stimulus-specific RBPs bound to RNA. At the protein level, most drRBPs did not change except for five proteins that show significant (*p*-value ≤0.05) changes in their abundance both at the protein and RBP levels post treatment (Additional file 2: Figure S1). Three of these five proteins, the calcium-binding EF hand protein, rotamase CYP1 and the RNA-binding protein (AT1G60650), show contrasting responses at protein abundance and RNA-binding levels i.e. increasing RNA-binding level and decreasing at protein abundance. Responses of two proteins, SWIB/MDM2 domain protein and stress inducible protein, show similar trends both at protein and RNA-binding levels. Overall, the data show that changes observed in RNA-binding to the protein are due to RBP-RNA interactions and therefore part of the cellular response to the stress stimulus.

### Domain organization of drought stress responsive RBPome

To further characterize the composition of the drRBPome, we looked at the RBD enrichment and noted that 50 proteins contain the RNA recognition motifs (RRM), representing the most prominent RBD in this dataset (Additional file 3: Figure S2). Six of the RRM containing proteins also contain the NTF2 domain. Other predominant “classical” RBDs include the zinc finger (ZF)-CCCH, K-homology, DEAD helicases, like-Smith (commonly know as LSm), poly(A) binding protein and cold shock domain (Additional file 3: Figure S2). Additionally, 300 proteins contain unknown or unconfirmed RBDs (Additional file 4: Table S2). The RDBs gave rise to a three-way classification. Category I comprises proteins linked to RNA biology based on their RBDs and/or role in RNA processing (42% of the RBPome), category II contains ribosomal proteins (5%) and category III contains proteins with currently unknown RNA-interactions (53%) (Fig. 1c). The later is indicative of a large set of potential RNA-interacting proteins that are yet to be fully characterized and in particular, their mode of action and target RNAs.

### The drRBPs shows enrichments in gene ontology (GO) categories

Perhaps not surprisingly, drRPBs are enriched in the functional categories, “nucleic acid binding” and “RNA binding” (Fig. 1d) and drought-specific processes, which are among the most enriched biological processes. The latter include “response to stress” and “response to stimulus” including hormonal and temperature stimulus, “response to ABA stimulus”, “response to osmotic stress” and “response to water deprivation” (Fig. 1e, Additional file 5: Table S3). In addition, signal transduction relay associated processes are also enriched including “transport” and “establishment of localization in cell”. In the latter, proteins enriched in this category include six NTF2 proteins that are involved in nucleocytoplasmic transport of mRNA, a process that enables translation of the respective mRNAs at their destination site [26]. All the six NTF2 proteins decrease in abundance within the first hour of treatment potentially signaling a reduction in nucleocytoplasmic transport of their target mRNAs. In contrast, nuclear pore anchor, a protein that mediates the transport of RNA and other cargo between the nucleus and the cytoplasm, increase in abundance. The nuclear pore anchor has been shown to be necessary for RNA homeostasis between the nucleus and cytoplasm and is required for e.g. flowering time and auxin signaling [27].

Co-expression analysis was performed on a selected set of the most up-regulated and/or down-regulated proteins and the top 300 co-expressed proteins for further characterization. Overall, co-expression analysis shows that among the most ranked proteins were a general bias towards proteins that are time specific upon stress treatment, although some proteins that are differentially regulated at 1 h and 4 h also existed (Additional file 6: Table S6). Examples of the latter include ILITHYA, which had 56 co-expressed proteins from the drRBP responsive proteins, of which 17 are up-regulated upon drought stress. Up-regulated proteins included classical drought stress responsive proteins such as CBP80, proteins involved in intermediary metabolism such as phosphofructokinase (AT1G20950) and carbohydrate binding like fold (AT3G62360) and various RNA binding proteins including NTF2 (AT1G13730), eIF2 gamma (AT1G04170) and ribosomal protein L4/L1 (AT3G09630). The second highly represented co-expressed protein is the nucleolar GTP-binding protein (AT1G50920) with 55 proteins that are drought stress responsive and 13 of these are differentially regulated. The third is adenine nucleotide alpha hydrolases-like super protein (AT5G54430) with 41 (of which 15 are differentially regulated) proteins, followed by guanylate-binding protein (AT5G46070) that showed 38 (of which 13 are differentially regulated) proteins. Among the least represented is the flowering time control (AT4G16280) with 15 proteins (five are differentially regulated). Besides their classical biological process of developmental role, proteins co-expressed with flowering time control protein are also involved in RNA binding. Proteins co-expressed with adenine nucleotide alpha hydrolases-like super protein show a bias towards enrichment of biological processes such as “response to stress” and “primary metabolic process”. Of interest to note is that all the enriched biological process of the selected proteins and their respective co-expressed ones are involved in RNA metabolic processes, translational activities or intermediary metabolism and functionally are biased towards RNA-binding. Co-expression analysis suggests that the drRBPs have a strong connectivity network and biotechnologically may be important targets towards improving tolerance to drought stress in crop plants.

### EnigmaRBPs detected responsive to drought stress

**Fig. 2:**
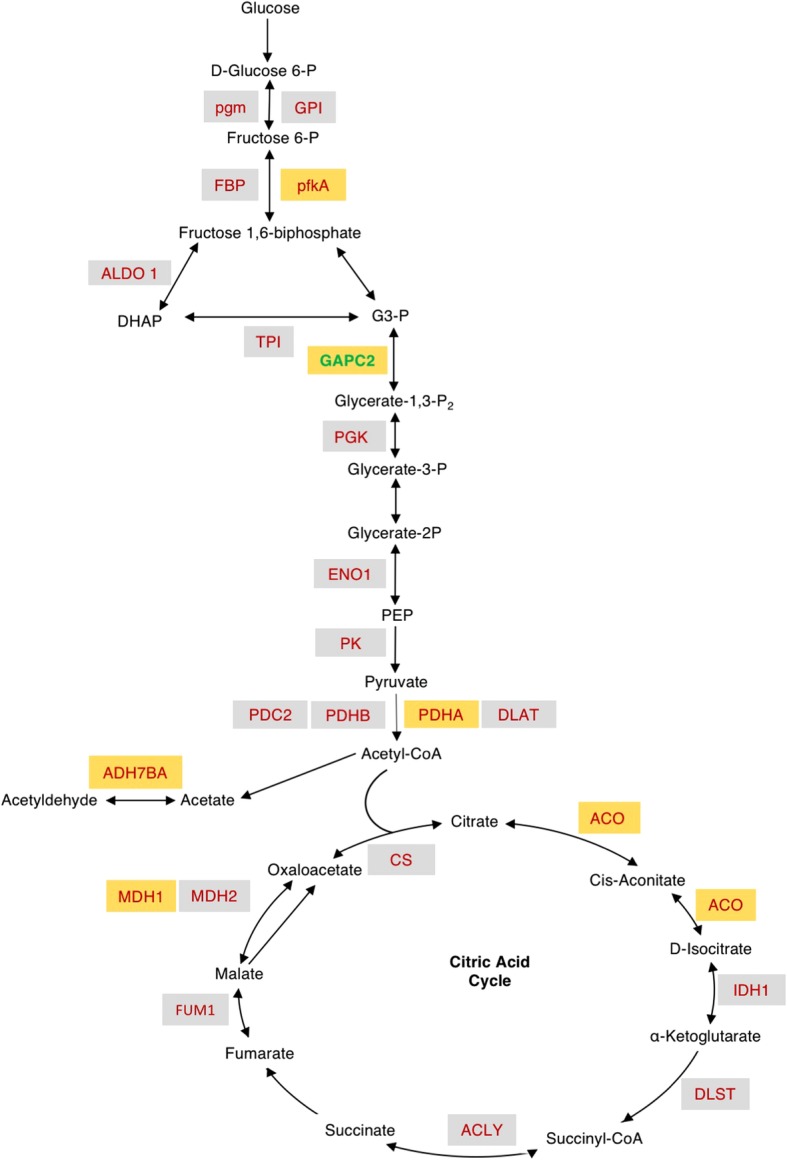
Schematic glycolytic and citric acid pathways illustrating the proteins identified responsive to drought stress at RBP level (orange boxes) with the bold green GAPC2 only identified in the current study and others including the enzymes in grey boxes have been previously identified [12]. Enzymes in orange boxes namely pfkA, GAPC2, ALDH7B4, ACO and MDH1 significantly increase and PDHA decrease in abundance after drought stress treatment (Additional file 1: Table S1). The figure is adapted from [12]). Abbreviations in order of appearance: pgm: phosphoglucomutase 2; GPI: glucose-6-phosphate isomerase; FBP: fructose 1.6-bisphosphate phosphatase or high cyclic electron flow 1; pfkA: phosphofructokinase 3; ALDO1: fructose bisphosphate aldolase 1; P: phosphate; TPI: triosephosphate isomerase; GAPC2: glyceraldehyde-3-phosphate dehydrogenase C2; P_2_: bisP; PGK: phosphoglycerate kinase; G3-P: glyceraldehyde 3-phosphate; ENO1: enolase; PK: pyruvate kinase; PDC2: pyruvate decarboxylase 2; PDHB: transketolase or pyruvate dehydrogenase E1 component beta subunit; PDHA: pyruvate dehydrogenase E1 component subunit alpha-3, chloroplastic; DLAT: mitochondrial pyruvate dehydrogenase subunit 2–2 or dihydrolipoyllysine-residue acetyltransferase component 2 of pyruvate dehydrogenase complex; ALDH7B4: aldehyde dehydrogenase 7B4; CS: citrate synthase; ACO: aconitase; IDH1: NADP-specific isocitrate dehydrogenase; DLST: dihydrolipoamide succinyltransferase; ACLY: ATP-citrate lyase; FUM1: fumarase 1; MDH1: cytosolic NAD-dependent malate dehydrogenase; MDH2: mitochondrial malate dehydrogenase 2

A pathway analysis of the unique UV-enriched and drRBPs was undertaken. The KEGG annotated pathways reveal a bias towards metabolic enzymes especially for proteins increasing in abundance after treatment. Seven stress-responsive proteins belong to the carbohydrate metabolism pathway (Additional file 7: Table S4) and six (glyceraldehyde 3-phosphate dehydrogenase C-2 (GAPC2), pyruvate dehydrogenase E1 component α-subunit (PDHA), phosphofructokinase, aldehyde dehydrogenase 7B4 (ALDH7B4), cytosolic NAD-dependent malate dehydrogenase 1 and aconitase 3 (ACO)) have a role in glycolysis and the citric acid cycles. Two proteins (ALDH7B4 and monodehydroascorbate reductase 1) are part of the ascorbate and aldarate metabolism (Fig. 2).

Identification of RBPs with a role in metabolism link post-transcriptional gene regulation to stress-induced metabolic changes and may suggest that RBPs exert their effect by (auto-)regulating their own or other mRNA species. Moreover, four of the carbohydrate metabolism proteins (GAPC2, ALDH7B4, PDHA and ACO) are also enriched in gene ontology categories “response to ABA stimulus”, “response to water derivation” or “response to oxidative stress”. In animals, besides their glycolytic activity, GAPC2 has non-glycolytic functions that depend on its subcellular localization, e.g. in the nucleus it acts as a signal for programmed cell death [28] and is involved in posttranscriptional regulation and maintenance of DNA integrity [29]. At protein level, GAPC2 expression has been observed to increase in response to cold stress [30]. In the present study, we note an increase of GAPC2 in response to drought stress at posttranscriptional level denoting a potential transcriptional rise of its target RNA. Aldehyde dehydrogenase 7B4, a member of the “turgor-responsive” ALDH genes [31] also increases in abundance after stress treatment. The ALDH protein family detoxifies aldehydes generated in plants when exposed to environmental stresses such as salinity and dehydration [32, 33]. Knockout mutants, *ALDH3I1* and *ALDH7B4* T-DNA, displayed higher sensitivity to dehydration and salinity stress compared to the wild-type plants consistent with a role of ALDH genes in stress responses [34]. At transcriptional level, abundance of *ALDH7B4* increases in plantlets and roots after dehydration and ABA treatments and declines in a time-dependent manner after stress relief [35]. In a previous study, we identified ALDH7B4 as a candidate RBP [12], and consistently, we find it enriched in the stress-responsive RBPome. It appears that ADLH7B4 has a dual function as a glycolytic enzyme and interacting with RNA thereby acting as a post-transcriptional gene regulator during drought stress. Another well-characterized glycolytic enzyme that we also noted to be drought stress responsive is ACO. Aconitase is an iron regulatory protein 1 (IRP1) that catalyzes the conversion citrate to isocitrate (Fig. 2). In animals, ACO1 is a bifunctional protein that becomes catalytically active in the presence of an iron-sulfur cluster in its catalytic center, while in the absence of the cluster, it operates as RBP, modulating the translation or stability of transcripts [36]. In plants, nitric oxide and oxidative stress have been shown to modulate the expression of ferritins [37] and to inactivate ACO catalytic activity [38] converting it to IRP1 through structural changes to its 4Fe-4S cluster. ACO3 is responsive to oxidative stress [39, 40] and interacts with mRNA in vivo [12]. The increase in abundance of ACO3 during drought stress is consistent with a post-transcriptional regulatory role that is likely to affect the transcriptome and eventually the proteome and metabolome during responses to stress. Taken together, it appears that drought stress-induced differential accumulation of RNA-interacting proteins is over-represented in specific functional groups.

### Biophysical characteristics and sequence topology of drRBPome

**Fig. 3:**
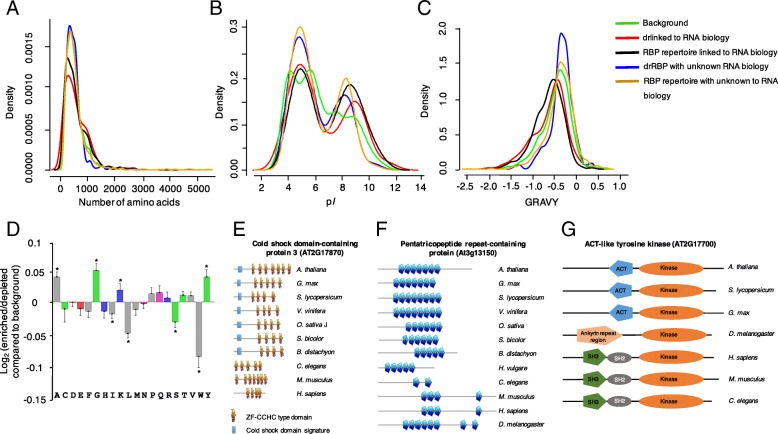
Biophysical features of drought stress responsive RBPome and domain conservation across species. Density of (**a**) protein length (number of amino acids), **b** isoelectric point (p*I*) and **c** hydrophobicity (gravy) were analyzed for RBPome responsive to drought stress with proteins linked to RNA biology (red), RBPome responsive to drought stress with proteins whose RNA biology is unknown (blue), RBP repertoire linked to RNA biology (black), RBP repertoire with unknown RNA biology (orange) [41] and input proteome from controls that are used as input or background (*N* = 5630) (green). Significances of differences between RBP subsets in (**a**-**c**) was tested using the Kolmogorov-Smirnov test. Compared to the reference data set, all the four subsets are significantly different number of amino acids (**a**), p*I* (**b**) and hydrophobicity (**c**) (*p* < 0.01), with the exception of number of amino acids in the RBP repertoire with unknown RBP biology. **d** Log_2_ enrichment of amino acid residues in the RBPome responsive to drought stress, determined using the composition profiler (http://www.cprofiler.org/). The significance of enrichment or depletion was tested by a two-sample T-test and amino acids that are significantly enriched or depleted (*p* ≤ 0.01) are marked with asterisks. **e**-**f** Domain copy numbers of the cold shock domain-containing protein 3 (**e**), the pentatricopeptide repeat-containing protein (**f**) and the ACT-like tyrosine kinase (**g**). The motifs and copy number assignments were performed using the ScanProsite (http://prosite.expasy.org/)

Biophysical and amino acid (aa) sequence characteristics were also analyzed to determine the physical properties that enable RBPs to interact with RNA. The drRBPome, much like the input reference and the RBP repertoire data [41], span the full spectrum of protein sizes, with the majority of proteins being < 1000 aa long (Fig. 3a). However, compared to the reference data, we notice that drRBPs linked to RNA biology behave the same as the RBP repertoire linked to RNA biology compared to the drRBP with unknown RNA biology and RBP repertoire with unknown RNA biology. Proteins linked to RNA biology show a high density for proteins with amino acid sequence length of between 1000 and 2000 compared to proteins whose RNA biology is unknown. A similar trend is noted on the isoelectric point (p*I*) distribution (Fig. 3b). The p*I*s of proteins enriched in RNA interaction show similar patterns distinct from the reference data. In addition, proteins with unknown RNA biology from both drRBP and RBP repertoire sets have the same configuration and the same for drRBP and RBP repertoire proteins linked to RNA biology. The p*I* distribution of the latter significantly shifts towards higher p*I* (≥8) as compared to the reference proteome. A slight hydrophobicity bias is noted on the proteins with unknown RNA biology compared to the proteins linked to RNA biology, however, the enhanced density peak for drRBP with unknown RNA biology could be attributed to the much smaller number of proteins in this data set (Fig. 3c). Overall, the consistent trend observed on the p*I*, number of amino acids and hydrophobicity distribution on proteins with unknown RNA biology compared to the proteins whose RNA biology are known, may suggest additional properties with implications in RNA interactions of the novel proteins in this set. If we consider overall aa frequencies in the drRBPome and input reference as the basis for the analysis, we note that aa residues with polar side chains are favored since they have high affinity for RNA such as lysine, which is significantly (*p* < 0.05) enriched. Additionally, glycine that interacts strongly with guanine [42], is also significantly enriched while aa with aliphatic side chains such as phenylalanine (F) and tryptophan (W) are generally underrepresented (Fig. 3d).

### Conservation of drRBPs across different species

Many of the drRBPs identified (85%, 127 proteins) have orthologs in other plants (notably in *Brachypodium distachyon*) (Additional file 8: Table S5) and 70% (101 proteins) have orthologs in human, mouse, drosophila, *Caenorhabditis elegans* and yeast hinting at ancient origin RBP-dependent responses. A comparative analysis of domain architectures reveals similarities and loss or gain of domain copies across different species. The Arabidopsis cold shock domain-containing protein 3 (AtCSD3), for example, has orthologs in nearly all organisms examined (Additional file 8: Table S5). AtCSD3 is the longest ortholog and similarly to mouse has seven ZF-CCHC- type domains. CSD3 contains glycine-rich regions and at least four ZF-CCHC-type domains (Fig. 3e). Importantly, in addition to the ZF-CCHC, the CSD domain is present and seems unique to plants and may have evolved to optimize survival under drought conditions that incidentally are also induced by freezing [43].

A pentatricopeptide repeat (PPR)-containing protein (At3g13150) showed higher PPR-repeat copies in plants and drosophila than in animals (Fig. 3f). PPR proteins are an emerging class of RBPs with a 35-aa motif, repeated in tandem up to 30 times and have been proposed to function as molecular adaptors for RNA processing [44]. RNA-binding selectivity is conferred by dimers where an AsnAsp (ND) interacts with uracil, AsnSer (NS) with cytosine, SN with adenine and ThrAsp (TD) with guanine [45].

The number of PPR-containing proteins in land plants is higher (> 450) as compared to algae, as well as protozoa, yeast or animals (< 50) [44]. Furthermore, PPRs have been reported to be involved in RNA metabolism in plant mitochondria and chloroplasts and are likely to have a regulatory role in the responses to abiotic stress [46]. It has also been demonstrated that mutations of PPR proteins can result in severe phenotypes due to disrupted expression of target genes, many of which are essential for plant survival (e.g. the Arabidopsis PPR mutant *high chlorophyll fluorescence (hcf)152* struggle to survive the seedling stage under autotrophic conditions due to defective carbon fixation [47]). These findings are therefore consistent with important functions of the RNA-binding PPR proteins in the adaptation to terrestrial environments.

Besides, CSD and PPR, which are protein already known to interact with RNA, we examined domain conservation among the most regulated proteins with no known RNA binding role. We noted a high degree of conservation in domains across species among the most highly up-regulated proteins including pyridoxal-dependent decarboxylase protein (AT5G11880), guanylate-binding protein (AT5G46070), rotamase CYP 1 (AT4G38740), serine-rich protein (AT5G25280). Similar observation has been made from the most down-regulated proteins including leucine-rich repeat protein (AT5G22320), calcium-binding EF hand protein (AT2G41100) and structural maintenance of chromosomes protein (AT3G54670), with the exceptions of ACT-like tyrosine kinase, also called serine/threonine/tyrosine kinase 8 (AT2G17700, Fig. 3g). The latter protein is implicated in chloroplast organization in addition to its protein phosphorylation role [48]. It contains a highly conserved kinase domain, which is common in all species. However, aspartate kinase, chorismate mutase and tyrosine A (ACT) domain is detected only in plant species, ankyrin domain only in drosophila and SH2 and 3 domains present in animal systems. The ACT domain is proposed to be a conserved regulatory binding fold that is linked to a wide range of metabolic enzymes that are regulated by amino acid concentration.

## Conclusions

In summary, this study characterizes systems level changes occurring in the RBPome during drought stress responses. It highlights that qualitative and quantitative changes in RBPome are likely to affect metabolic processes and carbohydrate metabolism in particular. Control and stability of metabolic processes during exposure to stress are known to increase survival thus implicating the significant changes in the RBPome in post-transcriptional mechanisms that enable regulatory plasticity essential for a timely stress response that in turn enhances short- and long-term adaptations. In addition, it turns out that RBPs have an important biological function during drought stress as changes in RBPs are indicative of a stress response signaling. Finally, our findings are also consistent with evolutionarily conserved roles of RBPs in post-transcriptional drought stress response mechanisms.

## Methods

### Cell culture and treatment

Cells derived from roots of *Arabidopsis thaliana* (ecotype Columbia-0) were grown in liquid medium, as previously described [39, 49, 50]. The cell cultures used in this study were obtained from Mrs. Xiaolan Yu in the Department of Biochemistry at the University of Cambridge. Cells were treated with 40% (*v*/v) polyethylene glycol (PEG) 6000, a dehydration-inducing agent to mimic drought stress or with equal volumes of media as a negative control. Three biological replicates of cells treated with PEG or mock-treated cells were collected at 1 h and 4 h post-treatment. Each time-point treatment has a corresponding mock treatment per replicate. The medium was drained using Stericup® filter unit (Millipore, Billerica, MA), and cells were rinsed with 1 × phosphate buffered saline immediately before UV-crosslinking [12].

### Abscisic acid (ABA) assay

Three biological replicates of cell suspension cultures for each time-point (controls at 0 h, 1 h and 4 h, and 40% PEG treated samples at 1 h and 4 h) were subjected to Phytodetek® ABA Immunoassay (Agdia Inc., Elkhart, Indiana, US) following the manufacturer’s instructions. ABA levels were measured and statistically evaluated between each control and treatment time-point.

### UV-crosslinking and interactome capture

In vivo UV-crosslinking and isolation of Arabidopsis RBPs was performed, as previously described [12], using a protocol that utilizes a modified method originally optimized for HeLa cells [11]. Sample from each time-point were split into two, one set for UV-crosslinking and the second set for non UV-crosslinking. Samples for UV-crosslinking were irradiated in vivo with UV (254 nm) and the mRNA-protein complexes were pulled down using oligo(dT) beads. Purified proteins were analyzed by label free tandem mass spectrometry. Similarly to [12], the quality of the mRNA-protein crosslinked complex pull-down was assessed by performing an additional control whereby the sample was treated with RNase T1/A mix (Thermo-Fisher Scientific) according to the manufacturer’s recommendations. To isolate RBPs, mRNA-protein samples were treated with RNase A/T1 mix to release them from the captured RNA molecules. Crosslinking and isolation of RBPs were evaluated by western blotting using antibodies against polypyrimidine tract-binding protein 1, β-actin (Sigma Aldrich, St Louis, MO, USA) and histone 3 (Abcam, Cambridge, UK) following manufacturer’s recommendations (see [12]).

### Protein digestion and mass spectrometry

Protein samples were reduced, alkylated, buffer exchanged and digested, as described elsewhere [12]. Dried peptides were resuspended in 20 μL of 5% (*v*/v) acetonitrile and 0.1% (v/v) formic acid and analyzed with Q-Exactive™ Hybrid Quadrupole-Orbitrap™ using nano-electrospray ionization (Thermo-Fisher Scientific, San Jose, CA) coupled with a nano-Liquid Chromatography (LC) Dionex Ultimate 3000 Ultra High Performance Liquid Chromatography (UHPLC) (Thermo-Fisher Scientific). Mass spectrometry parameters and run analysis were performed following the protocol described in [51].

### Mass spectrometry data analysis

Raw files were processed using the Proteome Discoverer v2.1 (Thermo-Fisher Scientific) interlinked with the local MASCOT server (Matrix Science, London, UK). MASCOT searches were carried out against *Arabidopsis thaliana* database (built using the Arabidopsis information resource (TAIR; release 10)) using a precursor mass tolerance of 20 ppm, a fragment ion mass tolerance of ±0.5 Da and trypsin specificity allowing up to two missed cleavages, peptide charges of + 2, + 3 and + 4. Carbamidomethyl modification on cysteine residues was used as a fixed modification, oxidation on methionine residues as variable modifications and the decoy database was selected. Further stringency was applied on the peptide spectrum matches (PSMs) by allowing “forward” and “decoy” searches by MASCOT to be re-scored using the Percolator algorithm in Proteome Discoverer v2.1 thus yielding a robust false discovery rate (FDR) of < 1%.

### UV-crosslink enrichment

Protein enrichment upon UV-crosslinking was performed as previously described [12] using Microsoft Excel. Proteins that were detected in both the UV-crosslinked samples and the control (non-UV crosslinked samples) were quantitatively analyzed to assess UV-crosslinking enrichment. Normalized intensities of UV-crosslinked samples were compared quantitatively against normalized intensities of the control (non-UV crosslinked samples), and a log_2_-fold change of ≥2 and *p*-value of ≤0.05 calculated using Student’s T-test corrected for multiple testing using a method described previously [52] were applied for proteins to be categorized as enriched RBPs and to be considered for further analysis.

### Drought stress responsive RB-proteome (drRBPome) analysis

After normalization of the data and UV-crosslink enrichment analysis, proteins from the UV-crosslink enrichment and those that were only identified in the UV-crosslinked samples were used for quantitative analyses. Proteins only detected in at least two biological replicates were included. In this analysis, samples collected at 1 h time-point, that is 1 h PEG treated samples and mock treated controls were compared against each other and similarly for samples collected at 4 h time-point. Proteins with a log_2_-fold change ≥1.5 and *p*-value ≤0.05 corrected for multiple testing a method detailed elsewhere (Benjamini and Hochberg [52]) represented the significantly responsive proteins and were categorized as the significantly regulated drought stress responsive RBPs (drRBPs).

### Bioinformatics analyses

#### Classification of RBPs and gene ontology analyses

Classical and non-classical RNA-binding domains (RBDs) were detected from the drRBPome identified both in this study using pfam (http://pfam.xfam.org; February 2017). RBPs and candidate RBPs were classified, as described previously [13]. Furthermore, three categories were extrapolated to give clarity to the data, as reported previously [19]. Category I contains all proteins that have been reported or shown to have a role in RNA associated processes (linked to RNA biology), category II comprises of all detected ribosomal proteins, and category III contains the remaining proteins that have either no known RBDs or known association with RNA. Gene ontology (GO) enrichments were performed using AGRIGO (http://bioinfo.cau.edu.cn/agriGO/) and pathway analysis was done with the KEGG mapper (http://www.kegg.jp/kegg/tool/annotate_sequence.html; February 2017), which annotates sequences by BlastKOALA. BlastKOALA is an internal annotation tool in KEGG that assigns KEGG Orthology numbers by BLAST searches against a non-redundant set of KEGG GENES using SSEARCH computation [53]. Co-expression for functional and data correlation analysis of selected up- and down- regulated proteins was performed using ATTED database (http://atted.jp).

#### Biophysical characteristics and sequence topographies analyses

Analyses of biophysical properties including length of proteins (number of amino acids), isoelectric points (p*I*) and hydrophobicity were performed using R (version 3.3.1). Amino acid composition enrichment between the drought stress responsive RBPome and input total proteome as reference as the background set was determined using the web-based composition profiler program (http://www.cprofiler.org/) using default setting and ordering amino acids by hydrophobicity (Kyte-Doolittle) and the significance level was further assessed using Bonferroni correction [54]. Length and sequences of amino acid were retrieved from TAIR (https://www.arabidopsis.org/tools/bulk/sequences/index.jsp), p*I* were obtained from TAIR (https://www.arabidopsis.org/tools/bulk/protein/index.jsp) and hydrophobicity values were calculated using the GRAVY calculator (http://www.gravy-calculator.de).

#### Evolutionary conservation of drRBPs

To understand the conservation and potentially, the role of drRBPs, InParanoid version 8 (http://inparanoid.sbc.su.se/cgi-bin/index.cgi, [55]) was used to identify their predicted orthologs among Arabidopsis*,* selected dicots (*Glycine max*, *Solanum lycopersicum*, *Vitis vinifera*), monocots (*Brachypodium distachyon*, *Hordeum vulgare*, *Oryza sativa*, *Sorghum bicolor*), *Saccharomyces cerevisae*, *Drosophila melanogaster*, *Caenorhabditis elegans*, *Mus musculus* and *Homo sapiens*. Here, a two-way prediction was possible and data was compiled in Excel. The InParanoid program generates ortholog groups including all inparalogs with scoring below 0.05, which is achieved by using clustering rules based on genome-wide pairwise sequence similarity matches between two species [55].

## Additional files

Additional file 1: mRNA-interacting proteins responsive to drought stress treatment at 1 h and 4 h treatment. (XLSX 103 kb)

Additional file 2: Proteins identified as responsive to polyethylene glycol treatment. (A) RNA binding protein (AT1G60650), (B) Rotamase CYP1 (AT4G38740), (C) SWIB/MDM2 domain protein (AT2G35605), (D) Calcium binding EF hand (At calmodulin like 4, AT2G41100), (E) Stress-inducible protein (AT1G62740). Total soluble protein changes are represented by the grey bars and RNA-binding protein or mRNA-interacting protein changes by the black bars. The asterisk represents significantly (*p* < 0.05) changing protein at a given time. (PDF 23 kb)

Additional file 3: Classical and non-classical RNA-binding domains in *Arabidopsis thaliana* drought stress responsive RBPs mined using pfam database. (A) Most represented classical RNA-binding domains. (B) Most represented non-classical RNA-binding domains. Bars in blue represent number of protein domains mined from differentially expressed drought responsive proteins compared to domains present in drought responsive time specific proteins in red. (PDF 33 kb)

Additional file 4: Protein domains of the drought stress responsive proteins extracted from the pfam database (http://pfam.xfam.org; February 2017). (XLSX 154 kb)

Additional file 5: Gene Ontology analysis of the significantly enriched drought stress responsive proteins performed using AgriGO software (http://bioinfo.cau.edu.cn/agriGO/). (XLSX 49 kb)

Additional file 6: Co-expression analysis of the most up - and/or down- regulated proteins mined using the ATTED database (http://atted.jp/top_search.shtml#GeneTable). (XLS 190 kb)

Additional file 7: KEGG BlastKOALA (https://www.kegg.jp/blastkoala/) pathways represented by the differentially abundant proteins responsive to drought stress (XLSX 44 kb)

Additional file 8: Inparalog and Orthologs clusters for drought stress responsive protein of *Arabidopsis thaliana* and selected organism mined using the Inparanoid database (http://inparanoid.sbc.su.se/). (XLSX 308 kb)

## Abbreviations

aa: Amino acid
ABA: Abscisic acid
ACO: Aconitase
ALDH: Aldehyde dehydrogenase
AtCSD3: *Arabidopsis thaliana* cold shock domain-containing protein 3
BLAST: Basic Local Alignment Search Tool
CBP80: Cap-binding protein 80 or ABA hypersensitive 1
D or Asp: Aspartate
drRB: Drought stress responsive RNA-binding protein
drRPPome: Drought stress responsive RNA-binding proteome
FDR: False discovery rate
Fe: Iron
GAPC2: Glyceraldehyde 3-phosphate dehydrogenase C-2
GO: Gene ontology
GR-RBP8: Glycine-rich RNA-binding protein 8
hr: Hour
IRP1: Iron regulatory protein 1
KEGG: Kyoto encyclopedia of genes and genomes
LC: Liquid chromatography
LSm: Like-Smith
MS/M: Tandem mass spectrometry
N or Asn: Asparagine
NTF2: Nuclear transport factor 2
PDHA: Pyruvate dehydrogenase E1 component α-subunit
PEG: Polyethylene glycol
p*I*: Isoelectric point
PPR: Pentatricopeptide repeat
PSMs: Peptide spectrum matches
RBD: RNA-binding domain
RBP: RNA-binding protein
RRM: RNA recognition motif
S or Ser: Serine
S: Sulphur
T or Thr: Tryptophan
TAIR: The Arabidopsis Information Resource
UHPLC: Ultra high performance liquid chromatography
UV: Ultra violet
*v*/v: Volume/volume
ZF: Zinc finger
