# Supplementary material for: Changes in the Arabidopsis RNA-binding proteome reveal novel stress response mechanisms
Source: BMC Plant Biol. 2019 Apr 11;19:139. doi: 10.1186/s12870-019-1750-x (PMC6460520; doi:10.1186/s12870-019-1750-x)
Supplement: Supplementary file 2 — Proteins identified as responsive to polyethylene glycol treatment. (A) RNA binding protein (AT1G60650), (B) Rotamase CYP1 (AT4G38740), (C) SWIB/MDM2 domain protein (AT2G35605), (D) Calcium binding EF hand (At calmodulin like 4, AT2G41100), (E) Stress-inducible protein (AT1G62740). Total soluble protein changes are represented by the grey bars and RNA-binding protein or mRNA-interacting protein changes by the black bars. The asterisk represents significantly (p < 0.05) changing protein at a given time. (PDF 23 kb) [file 12870_2019_1750_MOESM2_ESM.pdf]

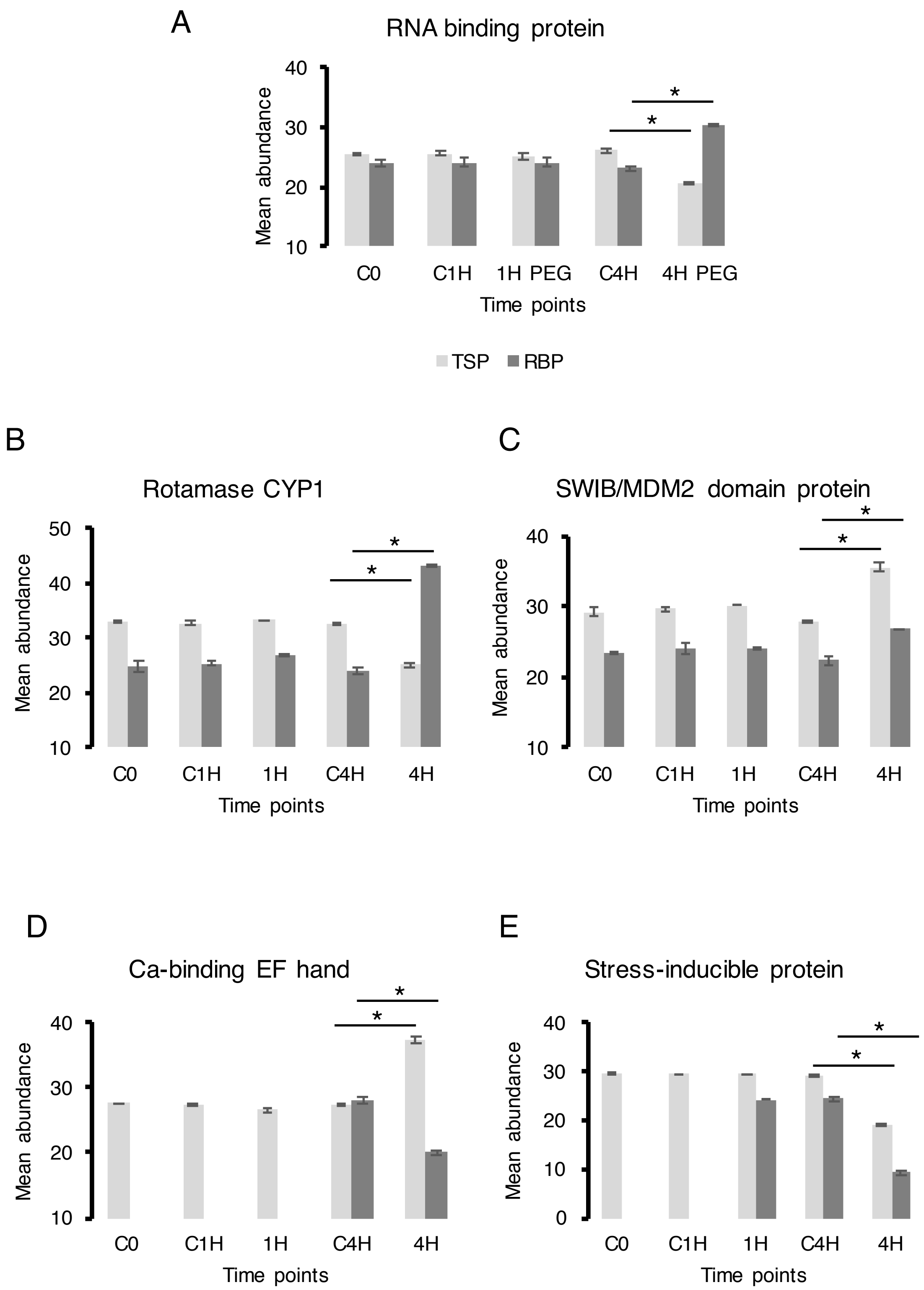
A
RNA binding protein
Mean abundance
40
30
20
10
*
*
C0
C1H
1H PEG
C4H
4H PEG
Time points
TSP
RBP
B
Rotamase CYP1
Mean abundance
50
40
30
20
10
*
*
C0
C1H
1H
C4H
4H
Time points
C
SWIB/MDM2 domain protein
Mean abundance
40
30
20
10
*
*
C0
C1H
1H
C4H
4H
Time points
D
Ca-binding EF hand
Mean abundance
40
30
20
10
*
*
C0
C1H
1H
C4H
4H
Time points
E
Stress-inducible protein
Mean abundance
40
30
20
10
0
*
*
C0
C1H
1H
C4H
4H
Time points
