# Supplementary figures and images for: Changes in the Arabidopsis RNA-binding proteome reveal novel stress response mechanisms
Source: BMC Plant Biol. 2019 Apr 11;19:139. doi: 10.1186/s12870-019-1750-x (PMC6460520; doi:10.1186/s12870-019-1750-x)

A

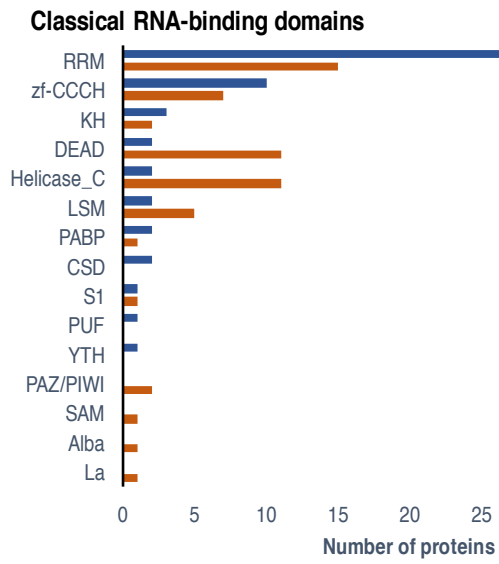

B

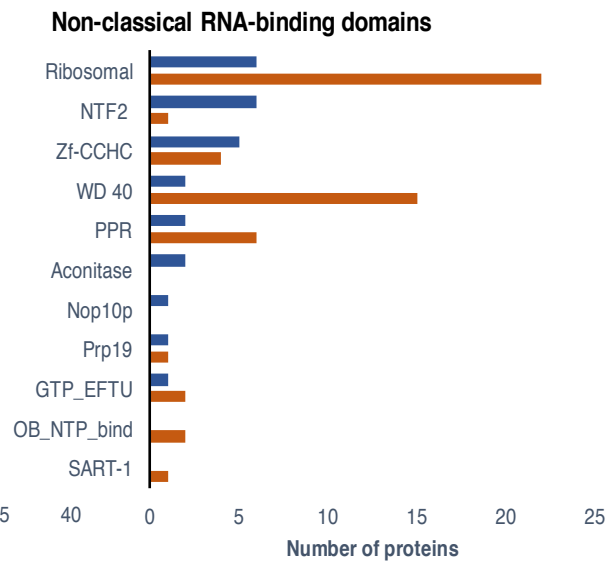

Supplement: Supplementary file 3 — Classical and non-classical RNA-binding domains in Arabidopsis thaliana drought stress responsive RBPs mined using pfam database. (A) Most represented classical RNA-binding domains. (B) Most represented non-classical RNA-binding domains. Bars in blue represent number of protein domains mined from differentially expressed drought responsive proteins compared to domains present in drought responsive time specific proteins in red. (PDF 33 kb) [file 12870_2019_1750_MOESM3_ESM.pdf]
